# Supplementary material for: The genome sequence of a cluster fly, Pollenia pediculata Macquart, 1834
Source: Wellcome Open Res. 2025 Apr 11;10:190. [Version 1] doi: 10.12688/wellcomeopenres.23961.1 (PMC12125572; doi:10.12688/wellcomeopenres.23961.1)
Supplement: Peer review history

# Reviewer report: Reviewer response for version 1

**DOI:** 10.21956/wellcomeopenres.26437.r123022

**Published:** 2025-05-30

**Authors:** Panagiotis Ioannidis

**Affiliations:** 1 Foundation for Research & Technology - Hellas, Crete, Greece

This manuscript describes the genome sequencing and assembly of Pollenia pediculata, a dipteran insect. The results show that the genome is of very good quality.

 Moreover, all sections are well-written but I particularly liked the introduction because the authors present a particularly challenging topic (i.e. the phylogenetic placement of Pollenia pediculata) that can be resolved with whole genome sequencing. Even though I know that the format for these papers is pretty well-defined, it would be nice if authors could optionally add a figure such as a species tree (pretty generic without a focus on any particular gene/gene family).

Are sufficient details of methods and materials provided to allow replication by others?

Yes

Is the rationale for creating the dataset(s) clearly described?

Yes

Are the datasets clearly presented in a useable and accessible format?

Yes

Are the protocols appropriate and is the work technically sound?

Yes

Reviewer Expertise:

Insect genomics, bioinformatics

I confirm that I have read this submission and believe that I have an appropriate level of expertise to confirm that it is of an acceptable scientific standard.

# Reviewer report: Reviewer response for version 1

**DOI:** 10.21956/wellcomeopenres.26437.r123019

**Published:** 2025-05-27

**Authors:** Arun Arumugaperumal

**Affiliations:** 1 Rajalakshmi Engineering College, Chennai, India

This is the first report of the whole genome sequence of *Pollenia pediculata* also known as cluster fly. The genome assembly is of size 1157 Mb. The mitogenome assembly is of size 15.82 kb. The genome is distributed in 6 chromosomes. 

 The data accession numbers mentioned in the data note is working fine. High-end sequencing technology was used to assemble the genome sequence. It is evident by the N50 value of 12.4 Mb. A BUSCO score of 99% indicates near completeness of the genome sequenced. The photograph showing the specimen organism is very clear.

 In page 3 change P. pendiculata to P. pediculata.

 The data note can be accepted for Indexing.

Are sufficient details of methods and materials provided to allow replication by others?

Yes

Is the rationale for creating the dataset(s) clearly described?

Yes

Are the datasets clearly presented in a useable and accessible format?

Yes

Are the protocols appropriate and is the work technically sound?

Yes

Reviewer Expertise:

Bioinformatics, Genomics

I confirm that I have read this submission and believe that I have an appropriate level of expertise to confirm that it is of an acceptable scientific standard.
